# New York Letters

**Published:** 1882-11

**Authors:** 


### ARTICLE XII.

NEW YORK, OCT. 14, 1882.

MESSRS. EDITORS:—There seems just now a great fever on the part of a large number in the profession to have the title *Professor.* In this city a new "post-graduate school" has been conceived, and is about to be delivered to the professional world. What the infant will amount to we can't tell. Its birth is expected early in November. By its advent, several hitherto unknown men will be launched into the world as professors, and perhaps the wailing and suffering public will be glad to know the merits of these professors. But this is not enough, for, by the opening of one more school, a few men have been left who do not own a medical college, and who cannot be styled professor by the admiring public; and as the title "doctor," as a mantle to cover their ignorance and wish for distinction, had become rather a common garment, they too want the fashionable cloak of professorship to cover the too thin but honorable vestment, doctor.

Therefore the times are pregnant with about-to-be-opened clinics and new style schools, wherein the few men not now connected with a medical school can be taught by these self-appointed and highly skilled professors. That these professors may have clinical material for the illustration of their knowledge, new and free hospitals and dispensaries are being opened and largely advertised to the public. To satisfy the demand for clinical material at these free treatment hospitals, the city of New York is thoroughly canvassed, and any deficiency supplied by bringing patients from the neighboring cities. What are we coming to in the near future? Perhaps in the near future a new line of steamers will be run by an organization of professors, bringing a supply of clinical material from London or Paris, or from the isles of the sea. However, before this enterprise is carried into effect, there will be a strong effort to use all sick persons in the city of New York and vicinity, and to "encourage home trade," a small sum will be paid every such patient (perhaps fifty cents or one dollar) at each time he appears for the illustration of the professor's skill and wisdom.

The title of professor being in such demand, would it not be "supplying a need long felt" to establish a school granting a diploma and conferring the title of professor, which means that the owner does not wish to advertise (in a manly way), and that he believes in the doctrine of professors, viz: that the big fish were created to eat the little fish. Then again, a new occupation for young men will be opened; for as professors increase, the supply of men to listen and admire at the clinics will be too small, therefore every professor will employ several listeners and admirers, paying them each a salary by the year to attend and learn at his clinic. It is to be hoped that the earnest enthusiasm now shown in the business of teaching each branch of medical science and art will not assume sufficient proportions to seriously affect the general prosperity of the land, by withdrawing so large a number of men from general manufactures. It is to be hoped, also, that there will remain in the medical profession a sufficient number of men who will devote themselves to earnest and honest study of professional duties, and thus keep up a line of succession of the true physician and surgeon or doctor. W.

MEDICAL TROUBLES IN MAINE.—No doubt but that our New York medical friend is right, and an echo of the same sentiment comes to us from Maine. It appears that the Maine General Hospital was foisted upon the State Medical Society; and now the country practitioners find that it attracts their paying patients, who go there to be operated upon, when they could be treated as well at home. The hospital is widely advertised. The same dissatisfaction is felt toward the Medical School of Maine. Attempts at remedying some of these defects will form the subject-matter of another meeting. H. D. V.

